# Effects of Substitution Position of Carbazole-Dibenzofuran Based High Triplet Energy Hosts to Device Stability of Blue Phosphorescent Organic Light-Emitting Diodes

**DOI:** 10.3390/molecules26092804

**Published:** 2021-05-10

**Authors:** Kyung Hyun Choi, Jae Min Kim, Won Jae Chung, Jun Yeob Lee

**Affiliations:** School of Chemical Engineering, Sungkyunkwan University, 2066, Seobu-ro, Jangan-gu, Suwon 16419, Korea; drchor@naver.com (K.H.C.); jmkim52@skku.edu (J.M.K.); wderwder@naver.com (W.J.C.)

**Keywords:** p-type host, blue device, phosphorescent device, lifetime, efficiency

## Abstract

High triplet energy hosts were developed through the modification of the substitution position of carbazole units. Two carbazole-dibenzofuran-derived compounds, 9,9′-(dibenzo[b,d]furan-2,6-diyl)bis(9*H*-carbazole) (26CzDBF) and 4,6-di(9*H*-carbazol-9-yl)dibenzo[b,d]furan (46CzDBF), were synthesized for achieving high triplet energy hosts. In comparison with the reported hole transport type host, 2,8-di(9*H*-carbazol-9-yl)dibenzo[b,d]furan (28CzDBF), 26CzDBF and 46CzDBF maintained high triplet energy over 2.95 eV. The device performances of the hosts were evaluated with electron transport type host, 2-phenyl-4, 6-bis(3-(triphenylsilyl)phenyl)-1,3,5-triazine (mSiTrz), to comprise a mixed host system. The deep blue phosphorescent device of 26CzDBF:mSiTrz with [[5-(1,1-dimethylethyl)-3-phenyl-1*H*-imidazo[4,5-b]pyrazin-1-yl-2(3*H*)-ylidene]-1,2-phenylene]bis[[6-(1,1-dimethylethyl)-3-phenyl-1*H*-imidazo[4,5-b]pyrazin-1-yl-2(3*H*)-ylidene]-1,2-phenylene]iridium (Ir(cb)_3_) dopant exhibited high external quantum efficiency of 22.9% with a color coordinate of (0.14, 0.16) and device lifetime of 1400 h at 100 cd m^−2^. The device lifetime was extended by 75% compared to the device lifetime of 28CzDBF:mSiTrz (800 h). These results demonstrated that the asymmetric and symmetric substitution of carbazole can make differences in the device performance of the carbazole- and dibenzofuran- derived hosts.

## 1. Introduction

The device performances of blue phosphorescent organic light-emitting diodes (PhOLEDs) are behind the red and green PhOLEDs [1,2,3,4,5,6]. As a result, the red and green PhOLEDs are already commercialized, blue is still a fluorescent emitter. The device performances of blue PhOLEDs are far behind that of the red and green PhOLEDs with respect to device operational time, especially. To replace a blue fluorescent emitter with a phosphorescent emitter, the development of a high triplet energy host should also be carried out.

Since progress in the device performance of blue PhOLEDs is the most effective way to improve the performance of full-color OLED panels, a lot of studies have been conducted to enhance the device performance of blue PhOLEDs. For device operational time, the reported studies offer a restricted number of device lifetime studies in the deep blue region. Due to a lack of stable deep blue phosphorescent emitter and stable high triplet energy hosts, most of the device lifetime studies for blue PhOLEDs were in the sky blue region [7,8,9,10,11]. As we have known, there are only three works of literature presenting device lifetime in the deep blue region with a y coordinate of below 0.2. The study using the Pt emitter from Li et al. showed a y coordinate of 0.18 and device lifetime of 800 h at 100 cd m^−2^, and a low external quantum efficiency (EQE) of 3.2% [12]. The other studies using an Ir emitter were conducted from our works. Exciplex device, oCBP:mSiTrz, exhibited y coordinate of 0.16 and device lifetime of 1900 h at 100 cd m^−2^, and a high EQE of 21.6% [13]. The electroplex device, Device II, achieved a y coordinate of 0.19 and device lifetime of 8500 h at 100 cd m^−2^, and a high EQE of 20.3%. The top-emitting electroplex device, Device III, accomplished y coordinate of 0.13 and device lifetime of over 10,000 h at 100 cd m^−2^, and high EQE of 27.6% [14].

In general, high triplet energy host materials are crucial to improve the device performances of highly efficient deep blue phosphorescence organic light-emitting diodes (PhOLEDs). The high triplet energy is essential when designing the host materials for blue PhOLEDs because the triplet excitons of the phosphors can be transferred to hosts if the hosts do not have higher triplet energy than the emitter [15]. Based on this principle, a lot of host materials have been synthesized using the high triplet energy building blocks [16,17,18,19,20,21,22,23,24,25,26,27,28]. Among the many building blocks, carbazole has high triplet energy and electron-rich characteristics leading to good hole transporting ability. Therefore, carbazole is one of the most common hole transport moieties in the design of the host materials [16,17,21,22,23,24,25,26,27]. Other than carbazole, dibenzofuran also has high triplet energy and bipolar carrier transport properties. However, the carrier transport property itself is not good enough, which forced the dibenzofuran to be modified with other functional moieties for improved charge transport property [18,19,20,28]. The dibenzofuran moiety has four substitution positions and the effect of substitution positions has been studied in several papers by modifying it with either hole transport or electron transport moieties [19,20]. However, the studies for the symmetrically or asymmetrically substituted dibenzofuran compounds with two functional moieties have been scarce. Therefore, we decided to study the effect of symmetric and asymmetric substitution of two carbazoles in the carbazole-dibenzofuran compounds on the photophysics and device characteristics of the blue PhOLEDs.

In this study, two p-type hosts, 9,9′-(dibenzo[b,d]furan-2,6-diyl)bis(9*H*-carbazole) (26CzDBF) and 4,6-di(9*H*-carbazol-9-yl)dibenzo[b,d]furan (46CzDBF), were derived from our previous work by changing the substitution position of carbazole [25]. As a reference host, 3,6-Bis(carbazole-9-yl)dibenzo[b,d]furan (28CzDBF), which was reported to be a high triplet energy host, was used since 28CzDBF has the same moieties but designed with different position of the carbazoles [29]. The two p-type hosts were mixed with 2-phenyl-4,6-bis(3-(triphenylsilyl)phenyl)-1,3,5-triazine (mSiTrz) to develop a P-N mixed host. The mixed hosts were evaluated as the hosts of deep blue-emitting [[5-(1,1-dimethylethyl)-3-phenyl-1Himidazo[4,5-b]pyrazin-1-yl-2(3*H*)-ylidene]-1,2-phenylene]bis[[6-(1,1-dimethylethyl)-3-phenyl-1*H*-imidazo[4,5-b]pyrazin-1-yl-2(3*H*)-ylidene]-1,2-phenylene]iridium (Ir(cb)_3_) phosphorescent emitter which was reported as a high efficiency deep-blue emitter in our previous work [30]. Among the mixed hosts, the mixed host of 26CzDBF:mSiTrz exhibited a high EQE of 21.3% with a color coordinate of (0.14, 0.16) and half luminance lifetime (LT50) over 1000 h at an initial luminance of 100 cd m^−2^. The LT50 of the 26CzDBF:mSiTrz device was extended by about twice that of 28CzDBF:mSiTrz device. This work demonstrated that the control of the substitution position can be an effective method for improving the operational time without sacrificing the advantages of the pristine compounds.

## 2. Results and Discussion

The three p-type hosts have a common structure with dibenzofuran core moiety substituted by two carbazoles. Two carbazoles were introduced at 2 and 6 positions in 26CzDBF, 4 and 6 positions in 46CzDBF, and 2 and 8 positions in 28CzDBF. The 28CzDBF host has high triplet energy over 2.90 eV as reported in the literature [25,29] and it was proven as the host for deep-blue PhOLEDs. The two hosts have differences in the substitution positions with 28CzDBF, which may make differences in photophysical characteristics and device performances. All three hosts have carbazoles having good hole-transport ability and work as the hole transport type (p-type) host. To satisfy the carrier balance, high efficiency, and small EQE roll-off in the blue PhOLEDs, the three hosts were mixed with an n-type host to compose a P-N mixed host system.

**Scheme 1:**
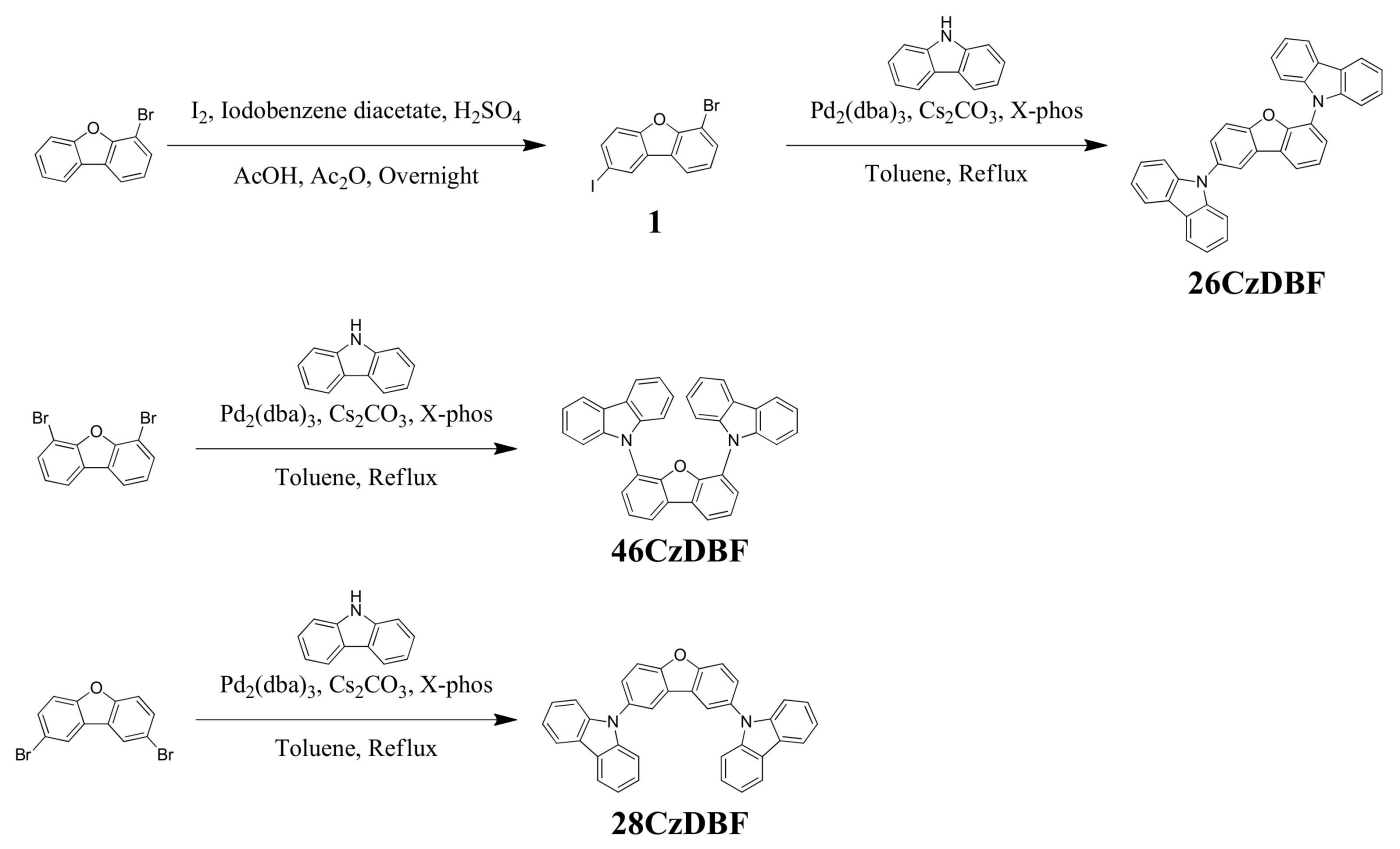
Brief synthetic scheme of 26CzDBF, 46CzDBF, and 28CzDBF.

The 26CzDBF, 46CzDBF, and 28CzDBF were synthesized by following the procedures shown in Scheme 1. An iodinated dibenzofuran intermediate (1) was prepared from 4-bromodibenzo[b,d]furan and then it went through Buchwald-Hartwig cross-coupling reaction with 9*H*-carbazole to produce 9,9′-(dibenzo[b,d]furan-2,6-diyl)bis(9*H*-carbazole) (26CzDBF). 4,6-dibromodibenzo[b,d]furan and 2,8-dibromodibenzo[b,d]furan went through Buchwald-Hartwig cross-coupling reaction with 9*H*-carbazole to produce 4,6-di(9*H*-carbazol-9-yl)dibenzo[b,d]furan (46CzDBF) and 2,8-di(9*H*-carbazol-9-yl)dibenzo[b,d]furan (28CzDBF), respectively. The final products were thoroughly purified by the purification processes of column chromatography and vacuum sublimation to remove residual solvents and impurities harmful to the device’s operational time. The purities of the final products were over 99.3% from high-performance liquid chromatography analysis. The final structures were confirmed by using chemical analytical methods such as ^1^H and ^13^C nuclear magnetic resonance spectrometer and mass spectrometer.

**Figure 1:**
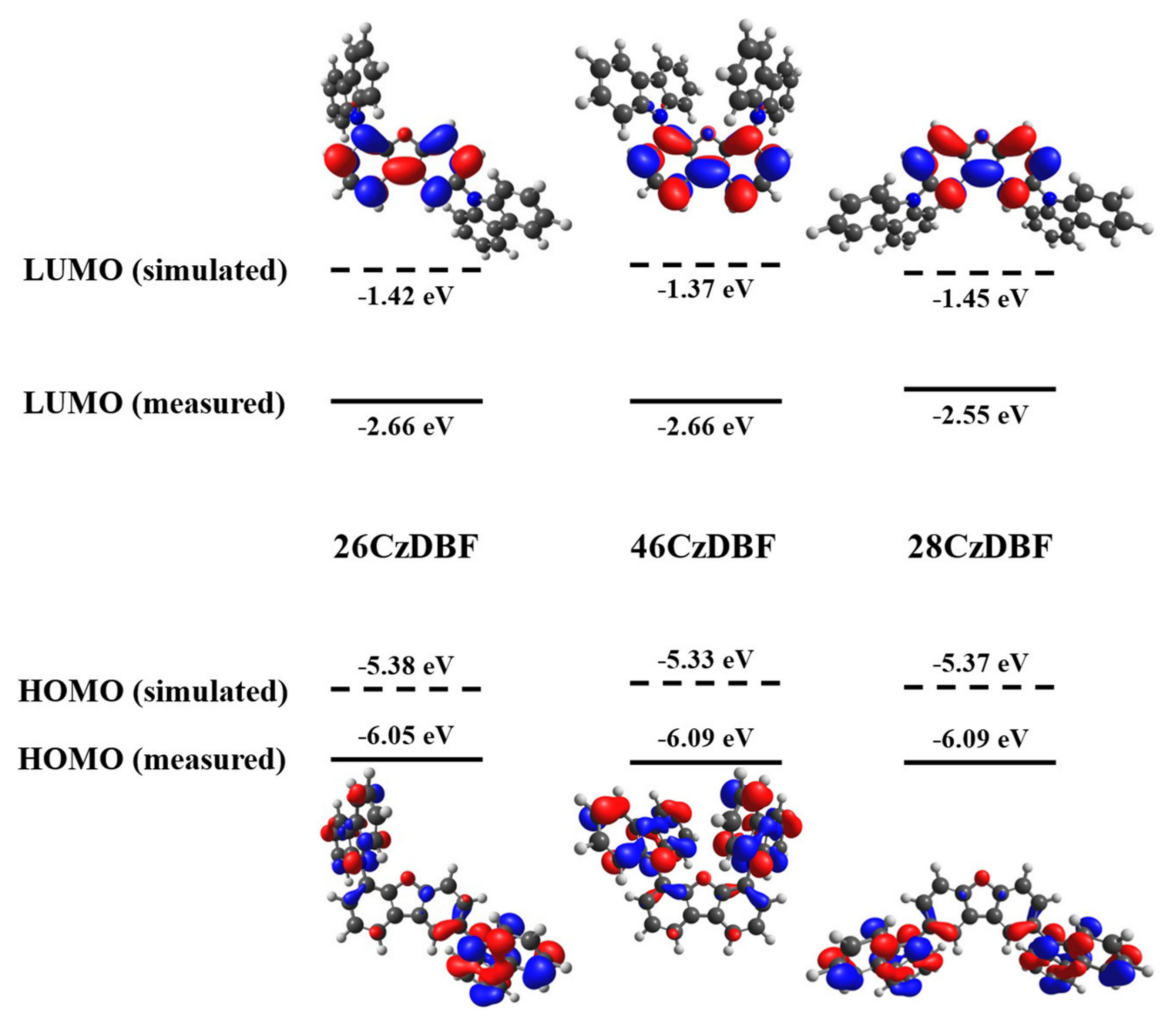
The HOMO and LUMO calculated and experimental results of 26CzDBF, 46CzDBF, and 28CzDBF.

Comprehensive electronic properties of the hosts were estimated by using the Gaussian 16 program based on the B3LYP 6-31G basis set. Highest occupied molecular orbital (HOMO), lowest unoccupied molecular orbital (LUMO), and the dihedral angles between the carbazoles and the dibenzofuran were calculated. In Figure 1, the HOMO and LUMO dispersions of 26CzDBF, 46CzDBF, and 28CzDBF are presented. All three compounds showed similar results in the HOMO and LUMO distributions. The HOMO was mostly dispersed on the carbazole and partially extended to the dibenzofuran. The LUMO was dispersed on the dibenzofuran dominantly. The dihedral angles between the carbazoles at 4-position and the dibenzofurans were 61.0° and 60.9° in 26CzDBF and 46CzDBF, respectively. The dihedral angles between the carbazole at 2-position and the dibenzofuran were 59.1° both in 26CzDBF and 28CzDBF. In consequence, the different substitution positions on dibenzofuran had little effect on the HOMO and LUMO dispersions and the dihedral angles between the carbazoles and the dibenzofuran.

The calculated HOMO and LUMO values were experimentally investigated by cyclic voltammetry (CV). Electrochemical oxidation and reduction processes were conducted and the data from the hosts are presented in Figure S1 in Supplementary Materials. The HOMO/LUMO energy levels of 26CzDBF, 46CzDBF, and 28CzDBF were −6.05/−2.66, −6.09/−2.66, and −6.09/−2.55 eV, respectively. The HOMOs were similar to each other because they are depended on electron-rich carbazoles.

**Figure 2:**
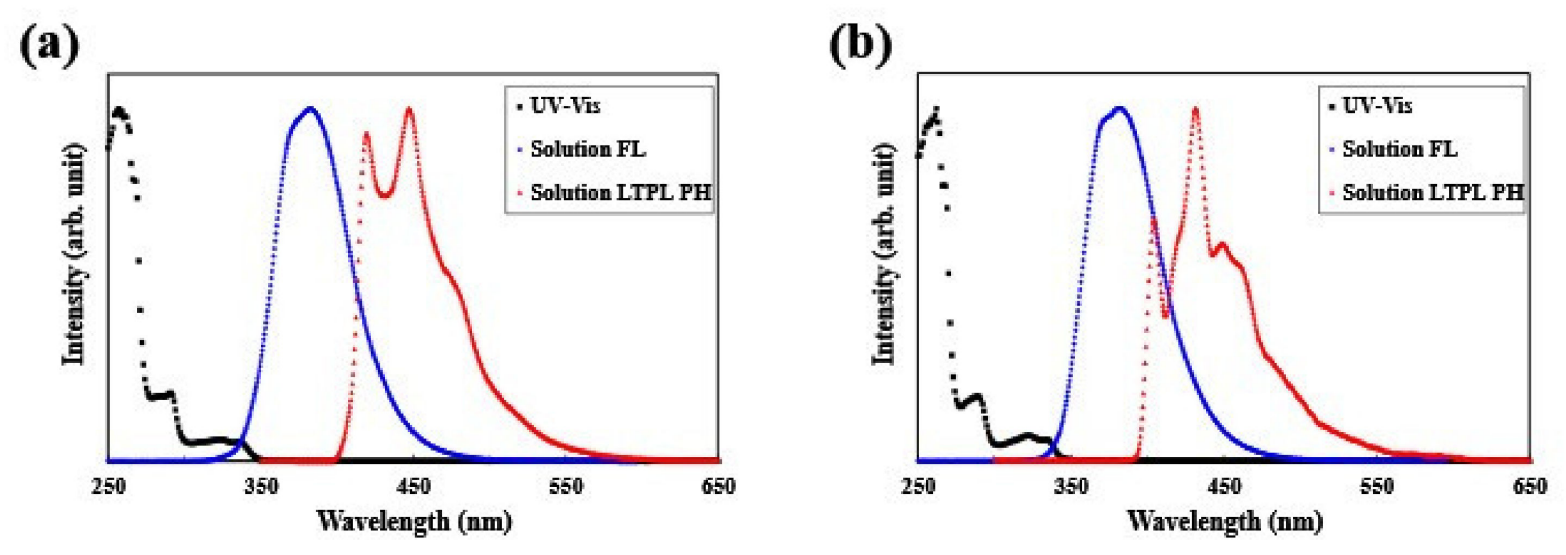
UV-Vis absorption spectra, fluorescent spectra (FL) and phosphorescent spectra (PH) of (**a**) 26CzDBF in solution and (**b**) 46CzDBF in solution.

Basic photophysical properties of 26CzDBF and 46CzDBF were analyzed to show their potential as the high triplet energy hosts for deep-blue phosphors. The ultraviolet-visible (UV-Vis), room temperature fluorescence, and low temperature (77 K) phosphorescence spectra were measured in dilute THF solution (1.0 × 10^−5^ M) (Figure 2). The UV-Vis absorption spectra of the 26CzDBF and 46CzDBF exhibited little discrepancy as a result of the different substitution positions. Strong UV-Vis absorption peaks below 300 nm corresponded to π-π* transitions of the carbazole units and the dibenzofuran unit, while the peaks between 300 nm and 350 nm were assigned to n-π* transitions of carbazole units. The optical energy band gaps of the hosts were calculated from the onset of the absorption edge. The optical band gaps were 3.56 eV (348 nm) for 26CzDBF and 3.60 eV (344 nm) for 46CzDBF.

Fluorescent emission peak wavelengths of 26CzDBF and 46CzDBF were 383 nm and 382 nm, and phosphorescent emission peaks were 419 nm and 420 nm, respectively. The singlet and triplet energies converted from the fluorescence and phosphorescence spectra were 3.24 eV and 2.96 eV for 26CzDBF, and 3.25 eV and 2.95 eV for 46CzDBF, respectively. The triplet energies were quite similar to that of 28CzDBF (2.98 eV). The results indicated that the two hosts would harvest the triplet excitons as the 28CzDBF host did previously. Thermal stabilities of the three hosts were analyzed by differential scanning calorimetry (DSC) for glass transition temperature (T_g_) and thermogravimetric analyzer (TGA) for decomposition temperature (T_d_). The T_g_s of the hosts were 133.2 °C, 113.6 °C, and 126.2 °C for 26CzDBF, 46CzDBF, and 28CzDBF, respectively. These high T_g_ values would prevent the emitting layer from local molecular motion. The decomposition temperatures (T_d_) were 456.7 °C, 412.4 °C, and 467.4 °C for 26CzDBF, 46CzDBF, and 28CzDBF, respectively. These high T_d_ values indicated that the hosts would endure the harsh condition of vacuum thermal evaporation without decomposition. The data from DSC and TGA are in Figure S2. All the material parameters of 26CzDBF, 46CzDBF, and 28CzDBF are summarized in Table 1.

**Figure 3:**
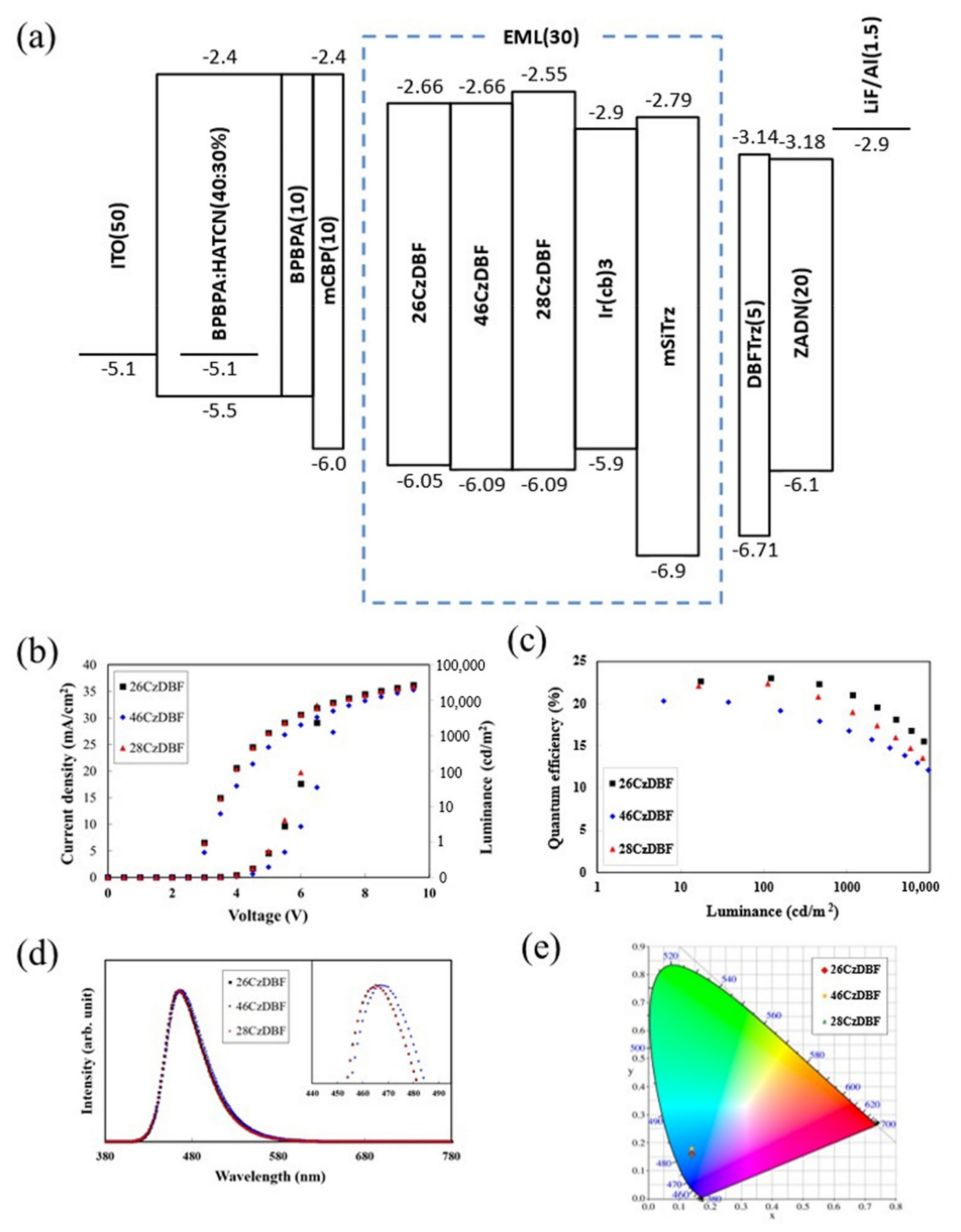
(**a**) Device structure, (**b**) Current density-voltage-luminance, (**c**) EQE-luminance, (**d**) EL spectra (inset: enlarged spectra), and (**e**) color coordinate of the 26CzDBF:mSiTrz, 46CzDBF:mSiTrz, and 28CzDBF:mSiTrz deep blue PhOLEDs.

As the triplet energies of the three hosts were over 2.95 eV, the hosts were evaluated with deep blue-emitting Ir(cb)_3_ emitter for high efficiency and long lifetime PhOLEDs. The device structure, current density-voltage, luminance-voltage, EQE-luminance, EL spectra, and color coordinate are presented in Figure 3. The chemical structures comprising the devices are in Figure S3. The current density and the luminance of the 26CzDBF and 28CzDBF devices exhibited much higher values than that of the 46CzDBF device at the same voltages. Driving voltages at 100/1000/10,000 cd m^−2^ of the devices were 3.9, 4.9, and 7.3 V in 26CzDBF and 28CzDBF, and 4.3, 5.4, and 8.1 V in 46CzDBF, respectively.

The EQE values of the devices were excellent for the EQE of the deep-blue PhOLED. The maximum EQE of the 26CzDBF device was 23.0% and the EQEs at 100 and 1000 cd m^−2^ were 22.9% and 21.3%, respectively. For the 46CzDBF device, the maximum EQE and the EQEs at 100 and 1000 cd m^−2^ were 20.3, 19.7, and 16.9%. For the 28CzDBF device, the maximum EQE and the EQEs at 100 and 1000 cd m^−2^ were 22.4, 22.4, and 19.5%, respectively. As the hole transporting ability of the 46CzDBF host was worse than that of the other hosts, the EQE of the 46CzDBF device was lower than those of the 26CzDBF and 28CzDBF devices. The red-shift of the EL spectrum might be related to the optical effect through the recombination zone. Considering the frontier orbital levels of the dopant and hosts, Ir(cb)_3_ acted as both electron trap and hole trap. For holes, the trap depth was 0.15–0.19 eV for a three p-type host. Since the trap depth was similar for all devices, the low current density of the 46CzDBF device could originate from the electrical properties of the host. The recombination zone of the devices might be mostly dependent on the carrier transport properties of the hosts. The relatively poor hole-transport property of the 46CzDBF host may shift the recombination zone to the hole transport layer side, which red-shifted the EL spectrum. The color coordinates were shifted from (0.14, 0.16) of the 26CzDBF and 28CzDBF devices to (0.14, 0.18) of the 46CzDBF device at 1000 cd m^−2^.

**Figure 4:**
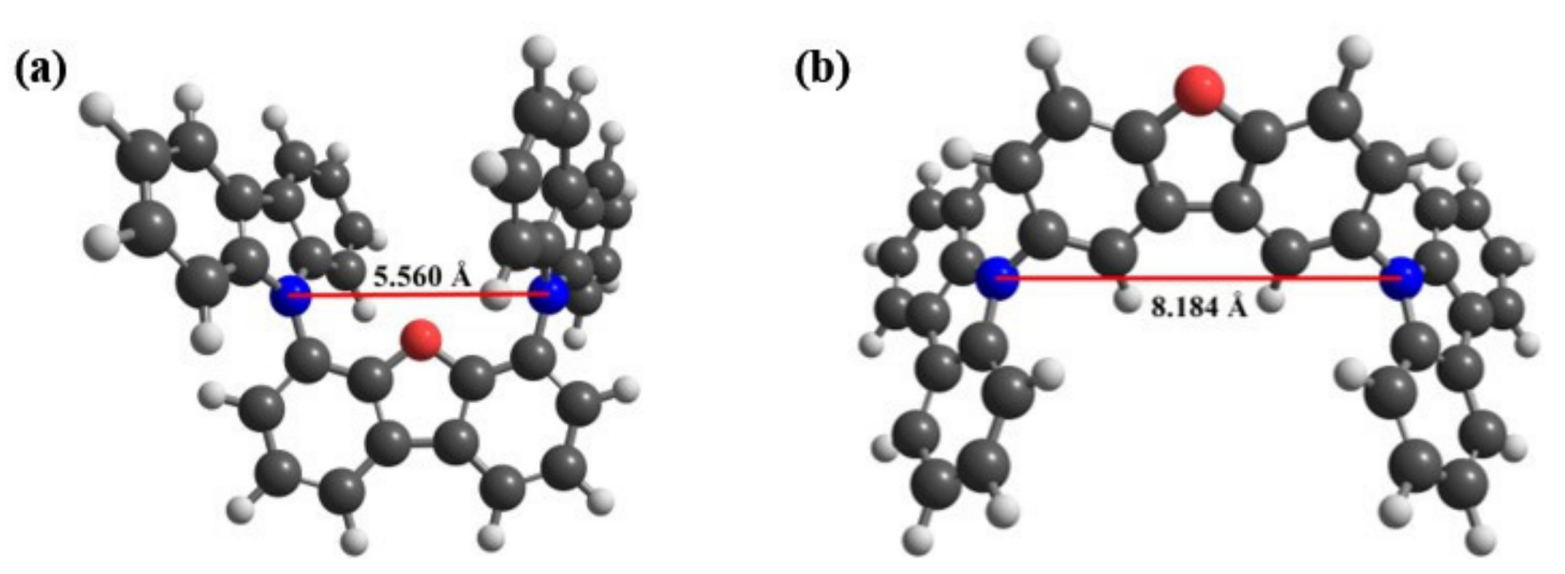
The calculated distance between the nitrogen atoms of the (**a**) 46CzDBF and (**b**) 28CzDBF.

**Figure 5:**
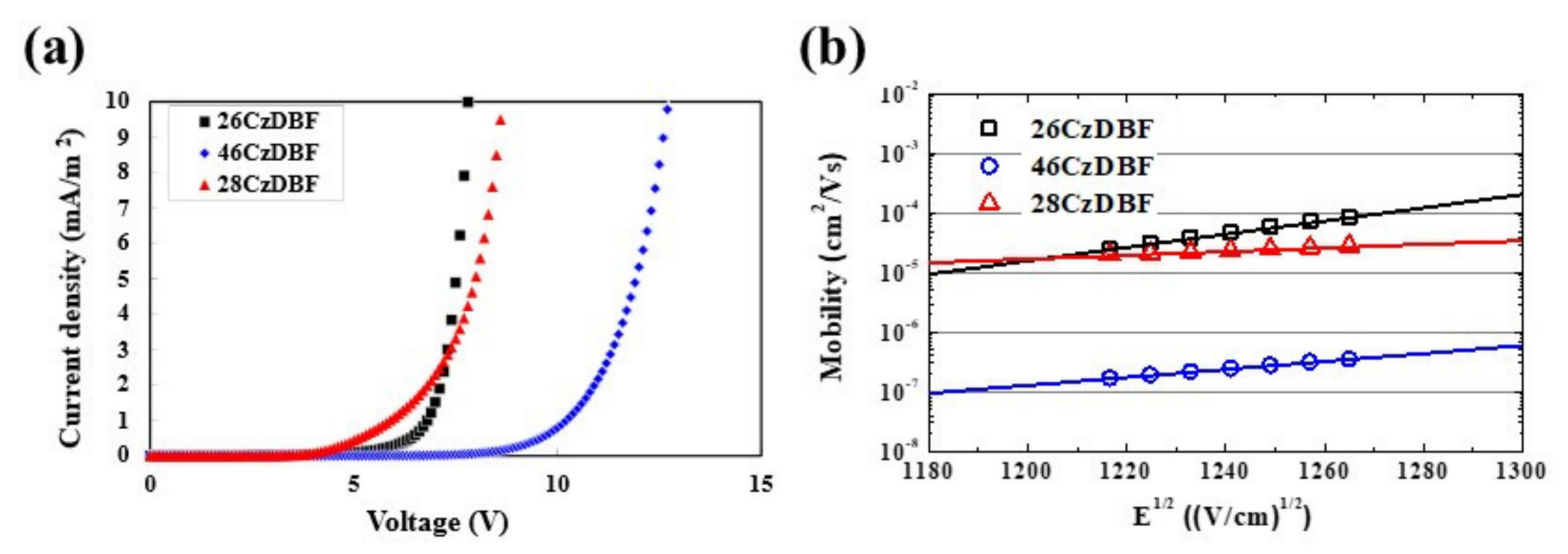
(**a**) Hole-only current density data and (**b**) hole mobility of 26CzDBF, 46CzDBF, and 28CzDBF hole-only devices.

The driving voltages of 46CzDBF were higher than those of the other devices even though the HOMO level of 46CzDBF and 28CzDBF was identical (−6.09 eV). The results could not be attributed to the difference in the HOMO levels of the hosts because the difference is only 0.04 eV. In this case, the main factor affecting the driving voltages may be the hole transporting ability of the hosts due to the difference in the molecular structure. The distance between two carbazole units is much closer in 46CzDBF than in 28CzDBF and 26CzDBF. The distance from one nitrogen atom to the other nitrogen atom is 5.560 Å in 46CzDBF and 8.184 Å in 28CzDBF as depicted in Figure 4. Since the host molecules transport carriers through the overlapped p-orbitals between host molecules, the sphere type molecular geometry of 46CzDBF hinders the hole transport [31]. Whereas, the extended molecular shape of the 28CzDBF and 26CzDBF is advantageous for extensive p-orbital overlap, facilitating the hole transport. The molecular shape with the short distance between the two carbazole units caused the high driving voltage of the 46CzDBF devices. This can be confirmed by the hole-only device (HOD) device data in Figure 5. The device structure of the HOD was ITO (50 nm)/PEDOT:PSS (60 nm)/hosts (50 nm)/Al (200 nm). The hole mobilities analyzed from the fitting of the space-charge-limited current of the HOD is depicted in Figure 5b. The hole mobilities at the root electric field of 1241 V^0.5^ cm^−0.5^ were 4.8 × 10^−5^ cm^2^ V^−1^s^−1^ for 26CzDBF, 2.5 × 10^−7^ cm^2^ V^−1^s^−1^ for 46CzDBF, and 2.4 × 10^−5^ cm^2^ V^−1^s^−1^ for 28CzDBF. The hole mobility of 46CzDBF was 192 times lower than that of 26CzDBF. From the current density-voltage and the hole mobility, the ability of the hosts to transport hole carriers can be evaluated, which confirmed that the 26CzDBF and 28CzDBF show much better hole-transport properties than 46CzDBF.

**Figure 6:**
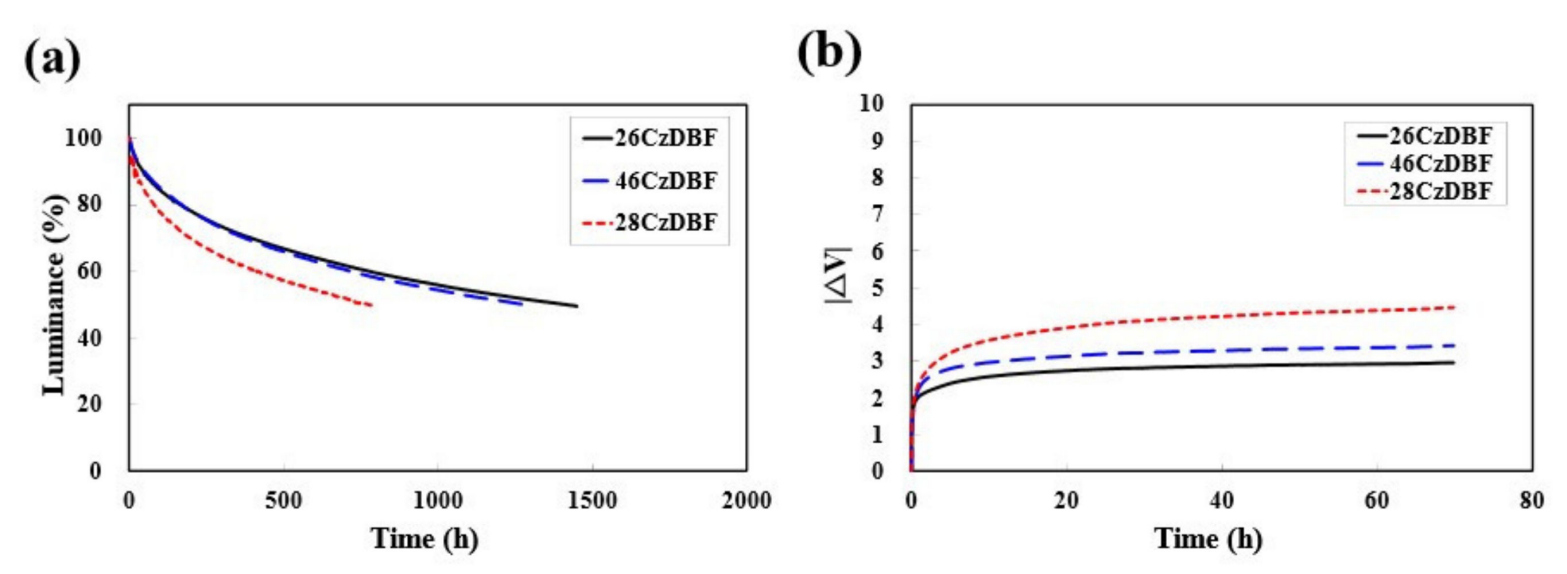
(**a**) Device lifetime data of the 26CzDBF:mSiTrz, 46CzDBF:mSiTrz, and 28CzDBF:mSiTrz deep blue PhOLEDs at an initial luminance of 100 cd m^−2^. (**b**) Voltage rise of HODs in the stability test at a current density of 2.5 mA cm^−2^.

Since device lifetime is the most challenging issue for deep blue PhOLEDs, the lifetime measurement of the deep-blue PhOLEDs was also carried out. In Figure 6, the device operational lifetime of the deep-blue PhOLEDs at an initial luminance of 100 cd m^−2^ is presented. The operational lifetime of the 26CzDBF and 46CzDBF devices up to 50% of initial luminance was over 1000 h. However, the lifetime of the 28CzDBF device was about 800 h. To examine the difference, the stability test of the HOD was carried out by stressing the HOD at a current density of 2.5 mA cm^−2^ because the p-type hosts mostly carry holes during the light emission process. The voltage rise during the hole stress test was traced because it reflects the resistance change of the device by the p-type host. Large voltage rise implies a degradation of the p-type host under positive polarons. In the hole stability test, the 26CzDBF and 46CzDBF hosts exhibited smaller voltage rises than the 28CzDBF host, indicating the good hole stability of the 26CzDBF and 46CzDBF hosts. This was well correlated with the device lifetime of the blue PhOLEDs, suggesting that the 2-/6- position or 4-/6- position substitution of dibenzofuran can stabilize the p-type host under positive polarons. Device performances including device lifetime are summarized in Table 2.

## 3. Conclusions

In conclusion, two hole-transport type hosts derived from carbazole and dibenzofuran were synthesized to study the effect of substitution position on the photophysical and electrical properties. Depending on the substitution position of the carbazoles on dibenzofuran, the hole transport properties were largely changed. The 4 and 6 position substitution of carbazole retarded the hole transport, but it stabilized the host under positive polarons. As a result, the blue PhOLEDs with the 26CzDBF host exhibited a high EQE of 22.9%, the deep-blue color coordinate of (0.14, 0.16), and the device lifetime of 1400 h at 100 cd m^−2^. This work unveiled that change in the substitution position of carbazole can make differences in the device performances of the carbazole- and dibenzofuran-derived hosts.

## Figures and Tables

**Table 1:** Summarized material parameters of 26CzDBF, 46CzDBF, and 28CzDBF.

	UV Edge [nm]	S_1_ ^(a)^ [eV]	T_1_ ^(b)^ [eV]	HOMO [eV]	LUMO [eV]	T_g_ ^(c)^ [°C]	T_d_ ^(d)^ [°C]
26CzDBF	348	3.24	2.96	−6.05	−2.66	133.2	456.7
46CzDBF	344	3.25	2.95	−6.09	−2.66	113.6	412.4
28CzDBF ^(e)^	353	3.28	2.98	−6.09	−2.55	126.2	467.4

^(a)^ Singlet energy was calculated from the peak wavelength of fluorescent emission. ^(b)^ Triplet energy was calculated from the peak wavelength of phosphorescent emission. ^(c)^ Glass transition temperature. ^(d)^ Decomposition temperature. ^(e)^ The photophysical and cyclic voltammetry data of 28CzDBF are referred to from previous works [25,29].

**Table 2:** Summarized device performances of deep blue PhOLEDs developed in this work compared with those of other devices in the literature.

Host	Dopant	100 cd m^−2^				1000 cd m^−2^		MAX	
		CIE (x, y)	LT_50_^(a)^	EQE	P.E ^(b)^	EQE	P.E	EQE	P.E
26CzDBF:mSiTrz	Ir(cb)_3_	0.14, 0.16	1400	22.9	23.5	21.3	17.3	23.0	27.9
46CzDBF:mSiTrz	Ir(cb)_3_	0.14, 0.18	1300	19.7	20.0	16.9	13.2	20.3	28.3
28CzDBF:mSiTrz	Ir(cb)_3_	0.14, 0.16	800	22.4	22.6	19.5	15.7	22.4	26.3
mCBP [12]	PtON1	0.14, 0.18	800	3.2		N/A		3.2	
mCBP [12]	PtNON	0.15, 0.21	17,000	9.7		8.2		9.8	
oCBP:pSiTrz [13]	Ir(cb)_3_	0.14, 0.16	1900	18.9	25.5	18.6	20.2	19.1	26.1
oCBP:mSiTrz [13]	Ir(cb)_3_	0.14, 0.16	1900	21.4	28.6	21.1	21.9	21.6	29.3
oCBP:DBFTrz [13]	Ir(cb)_3_	0.14, 0.18	500	15.4	22.5	14.4	17.0	15.5	22.8
mCBP:SiCzTrz (Device I) [14]	Ir(cb)_3_	0.14, 0.19	5900					15.6	
mCBP:SiCzTrz (Device II) [14]	Ir(cb)_3_	0.14, 0.19	8460	20.2		18.6		20.3	
mCBP:SiCzTrz (Device III) ^(c)^ [14]	Ir(cb)_3_	0.12, 0.13	10,700	27.6		25.6		27.6	
oCBP:CNmCBPCN [32]	Ir(CNpi)_3_	0.15, 0.25	366 ^(d)^			17.9	22.6	18.8	30.3
mCBP [33]	Ir(CNpi)_3_	0.15, 0.24 ^(e)^	9.7 ^(f)^			10.1 ^(e)^	9.0 ^(e)^	12.8	14.5
mCBP:BM-A10 [34]	FCNIr	0.15, 0.21 ^(g)^	1 ^(h)^	23	37	21	26	24	41
CNIm/CNCzCN1 [35]	CNIm	0.15, 0.26	130 ^(i)^			13.7	16.2	17.7	33.1

^(a)^ Device lifetime at an initial luminance of 100 cd m^−2^ up to 50% of initial luminance; ^(b)^ Power efficiency (lm W^−1^); ^(c)^ Top-emitting device; ^(d)^ Device lifetime at an initial luminance of 100 cd m^−2^ up to 70% of initial luminance; ^(e)^ Device data at a luminance of 500 cd m^−2^; ^(f)^ Device lifetime at an initial luminance of 500 cd m^−2^ up to 50% of initial luminance; ^(g)^ Color coordinate at a current density of 10 mA cm^−2^; ^(h)^ Device lifetime at an initial luminance of 400 cd m^−2^; ^(i)^ Device lifetime at an initial luminance of 200 cd m^−2^.

## Data Availability

Not applicable.

## Supplementary Materials

The following are available online, details of experiments and materials synthesis, Figure S1: Oxidative and reductive cyclic voltammetry voltage scan of 26CzDBF and 46CzDBF, Figure S2: Differential scanning calorimeter heating scan (a) and thermogravimetric heating scan (b) data of 26CzDBF, 46CzDBF, and 28CzDBF at a heating rate of 10 °C/min under nitrogen, Figure S3: The device structures of the deep blue PhOLEDs and chemical structures of the used materials.

## Sample Availability

Samples of the compounds are not available from the authors.
