# Supplementary material for: Effects of Substitution Position of Carbazole-Dibenzofuran Based High Triplet Energy Hosts to Device Stability of Blue Phosphorescent Organic Light-Emitting Diodes
Source: Molecules. 2021 May 10;26(9):2804. doi: 10.3390/molecules26092804 (PMC8126063; doi:10.3390/molecules26092804)
Supplement: Supplementary file 1 [file molecules-26-02804-s001.zip › molecules-1197472-SI.pdf]

Supporting information

Experimental

General information

All chemicals and reagents were purchased from commercial suppliers and were used as received. 4-Bromodibenzo[*b,d*]furan and 2,8-dibromodibenzo[*b,d*]furan were purchased from GOM Technology CO., LTD. 4,6-Dibromodibenzo[*b,d*]furan was purchased from SY innovation. 9*H*-carbazole was purchased from Sigma Aldrich Co.. Iodine was purchased from TCI Chem. Co.. Iodobenzene diacetate was purchased from Alfa Aesar. Acetic acid, acetic anhydride, sulfuric acid, and cesium carbonate were purchased from Daejung Chemical & Metal Co.. Tris(dibenzylideneacetone)dipalladium(0) ($Pd_2(dba)_3$) and X-phos were purchased from P&H Tech Co..

The ^{1}H and ^{13}C nuclear magnetic resonance (NMR) spectra were recorded using Unity Inova (Varian, 500 MHz) spectrometer. The LUMO levels were estimated using cyclic voltammetry (Ivium Tech., Iviumstat) scan data of the materials. The UV-vis spectra were measured by UV-vis spectrophotometer (JASCO, V-730) and the PL spectra were obtained by fluorescence spectrophotometer (PerkinElmer, LS-55). THF solution was used to dissolve the samples for the UV-vis and PL spectra measurement. The triplet energies of the materials were measured in THF solution at 77 K under liquid nitrogen. The instrument to measure mass spectra was an Advion, ExpresionL CMS spectrometer in APCI mode.

Synthesis

Synthesis of 6-bromo-2-iododibenzo[*b,d*]furan (1)

1 was synthesized according to the synthetic method from the literature procedure.[1]

Synthesis of 9,9'-(dibenzo[*b,d*]furan-2,6-diyl)bis(9*H*-carbazole) (26CzDBF)

1 (1.0 g, 3.1 mmol) was mixed with 9*H*-carbazole (1.2 g, 7.4 mmol), $Pd_2(dba)_3$ (0.6 g, 0.6 mmol), cesium carbonate (4.0 g, 12.3 mmol), and X-phos (0.6 g, 1.2 mmol) in a 100 mL round-bottomed flask. Toluene (30 mL) was poured into the flask and then the mixture was refluxed overnight under a nitrogen atmosphere. The reaction mixture was extracted using dichloromethane (MC) and deionized water. The organic portion was dried over magnesium sulfate and filtrated under reduced pressure. The concentrated crude mixture was purified by silica column chromatography using n-hexane/MC as an eluent. The 26CzDBF was obtained as a white power (1.0 g, 2.0 mmol). For further purification, vacuum sublimation was conducted. Finally, 26CzDBF was obtained as a white powder (0.7 g, 1.4 mmol).

^{1}H NMR (500 MHz, $CDCl_3$): δ 8.219 (d, 2H, J=7.7 Hz), 8.196-8.173 (m, 3H), 8.056 (dd, 1H, J=7.7, 0.9 Hz), 7.732 (dd, 1H, J=7.7, 1.3 Hz), 7.653-7.636 (m, 1H), 7.610-7.560 (m, 2H) 7.448-7.287 (m, 12H). ^{13}C NMR (125 MHz, $CDCl_3$): δ 155.29, 152.41, 141.70, 141.08, 133.38, 127.49, 127.20, 126.52, 126.27, 126.25, 125.78, 124.27, 123.92, 123.51, 122.66, 120.73, 120.63, 120.61, 120.48, 120.20, 113.68, 110.44, 109.80. MS (m/z): found, 498.1736 ([FAB]+); Calcd. for $C_{36}H_{22}N_2O$, 498.1732.

Synthesis of 4,6-di(9*H*-carbazol-9-yl)dibenzo[*b,d*]furan (46CzDBF)

46CzDBF was synthesized according to the synthetic method of 26CzDBF. The reactant

in the synthesis was changed from 1 to 4,6-dibromodibenzo[*b,d*]furan. The reaction mixture was purified with the same method of 26CzDBF. 46CzDBF was obtained as a white power (0.8 g, 1.6 mmol).

^{1}H NMR (500 MHz, $CDCl_3$): δ 8.189 (dd, 1H, J=7.6, 1.3 Hz), 7.995 (d, 2H, J=8.0 Hz), 7.654 (dd, 1H, J=7.8, 1.5 Hz), 7.607 (t, 1H, J=7.6 Hz), 7.280-7.250 (m, 2H), 7.201-7.155 (m, 4H). ^{13}C NMR (125 MHz, $CDCl_3$): δ 151.68, 140.97, 127.42, 126.87, 126.05, 124.41, 123.78, 122.97, 120.54, 120.27, 120.08, 110.21. MS (m/z): found, 498.1736 ([FAB]+); Calcd. for $C_{36}H_{22}N_2O$, 498.1732.

Synthesis of 2,8-di(9*H*-carbazol-9-yl)dibenzo[*b,d*]furan (28CzDBF)

28CzDBF was synthesized according to the synthetic method from the literature procedure.[2]

Device fabrication and measurements

The stack structure of the blue devices was ITO (50 nm)/BPBPA:HATCN (40 nm:30%)/BPBPA (10 nm)/mCBP (10 nm)/emitting layer (30 nm)/DBFTrz (5 nm)/ZADN (20 nm)/LiF (1.5 nm)/Al (200 nm). The emitting layers of the devices were $Ir(cb)_3$ doped 26CzDBF:mSiTrz, 46CzDBF:mSiTrz, and 28CzDBF:mSiTrz. The doping concentration of $Ir(cb)_3$ for the devices was 20 wt% and the host composition was 50:50. Prior to the optimized device fabrication, the emitting layer structure was optimized by changing the $Ir(cb)_3$ doping concentrations (20 and 30 wt%) using the $Ir(cb)_3$ doped 26CzDBF:mSiTrz, 46CzDBF:mSiTrz, and 28CzDBF:mSiTrz emitting layer. The abbreviations used for the layers are as follows. ITO is indium tin oxide,

BPBPA is N,N,N',N'-tetra[(1,10-biphenyl)-4-yl]-(1,10-biphenyl)-4,4’-diamine, HATCN is 1,4,5,8,9,11-hexaazatriphenylene hexacarbonitrile, mCBP is 3,3'-di(9*H*-carbazol-9-yl)-1,1'-biphenyl, mSiTrz is 2-phenyl-4,6-bis(3-(triphenylsilyl)phenyl)-1,3,5-triazine, DBFTrz is 2,8-bis(4,6-diphenyl-1,3,5-triazin-2-yl)dibenzo[*b,d*]furan, and ZADN is 2-[4-(9,10-Dinaphthalen-2-yl-anthracene-2-yl)-phenyl]-1-phenyl-1*H*-benzimidazole.
All layers of the devices in the experiments were deposited by vacuum thermal evaporation under high pressure of 3.0×10^{-7} torr. The materials were thermally evaporated and then the devices were encapsulated using a glass cover in the nitrogen-filled glove box to protect moisture and oxygen. The electrical characterization of the devices was performed using a Keithley 2400 source meter and optical characteristics were analyzed using a CS 2000 spectroradiometer. The device lifetime of the devices was evaluated at an initial luminance of 100 cd m^{-2} under a constant current driving condition.

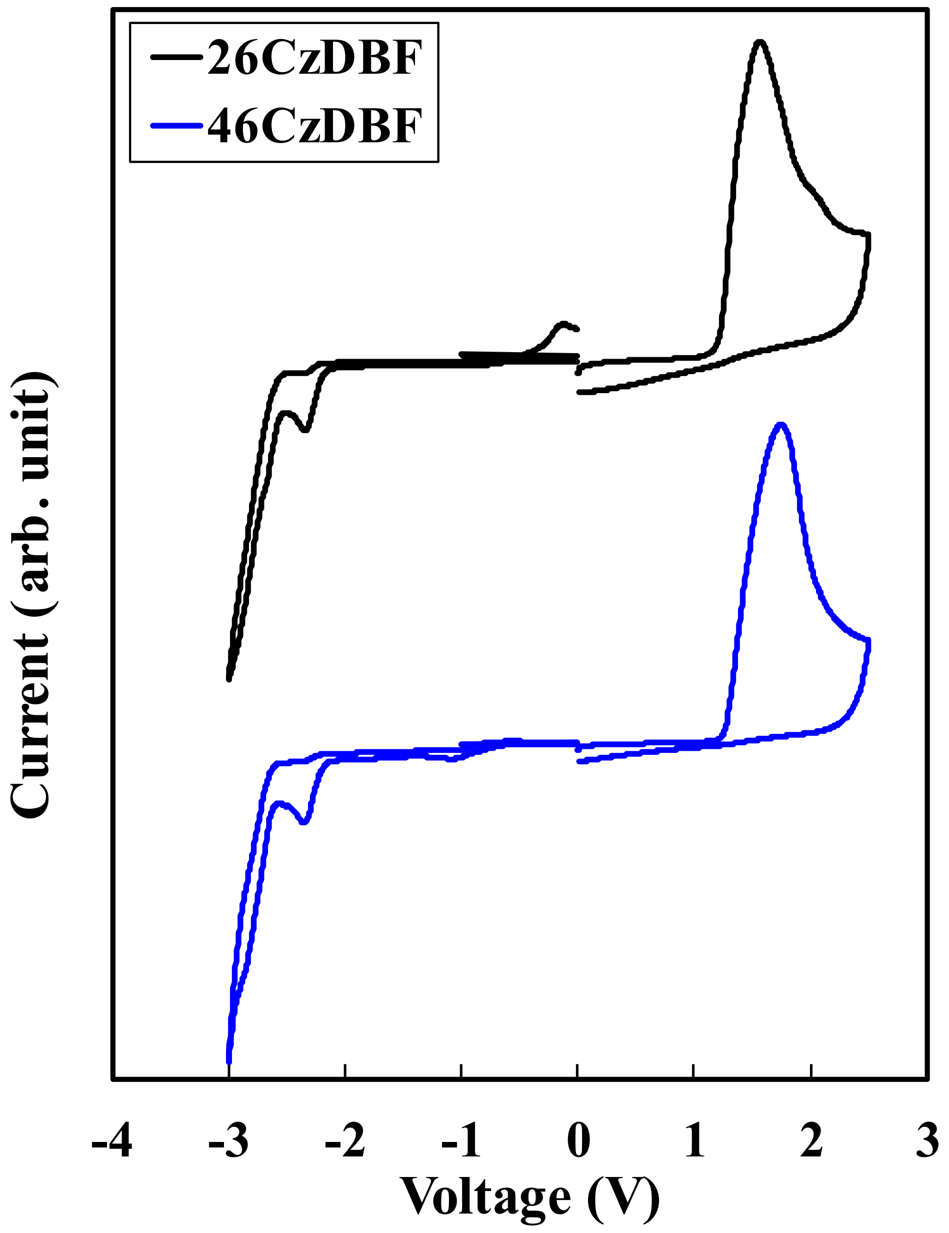

Figure S1. Oxidative and reductive cyclic voltammetry voltage scan of 26CzDBF and 46CzDBF.

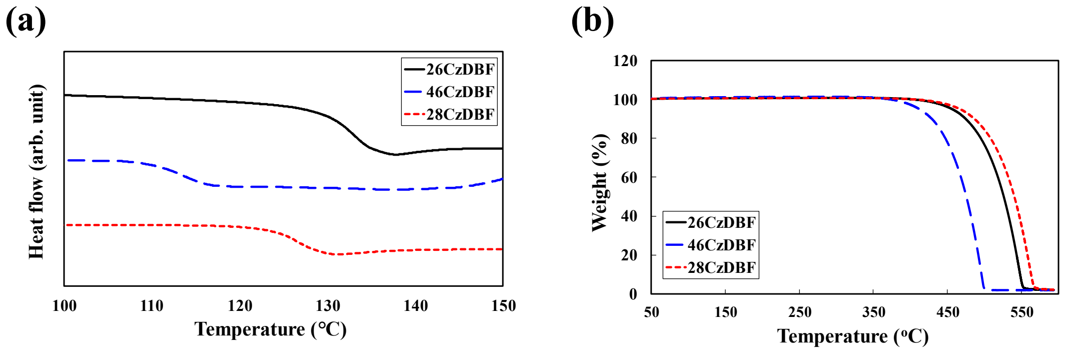

Figure S2. Differential scanning calorimeter heating scan (a) and thermogravimetric heating scan (b) data of 26CzDBF, 46CzDBF, and 28CzDBF at a heating rate of 10 °C/min under nitrogen.

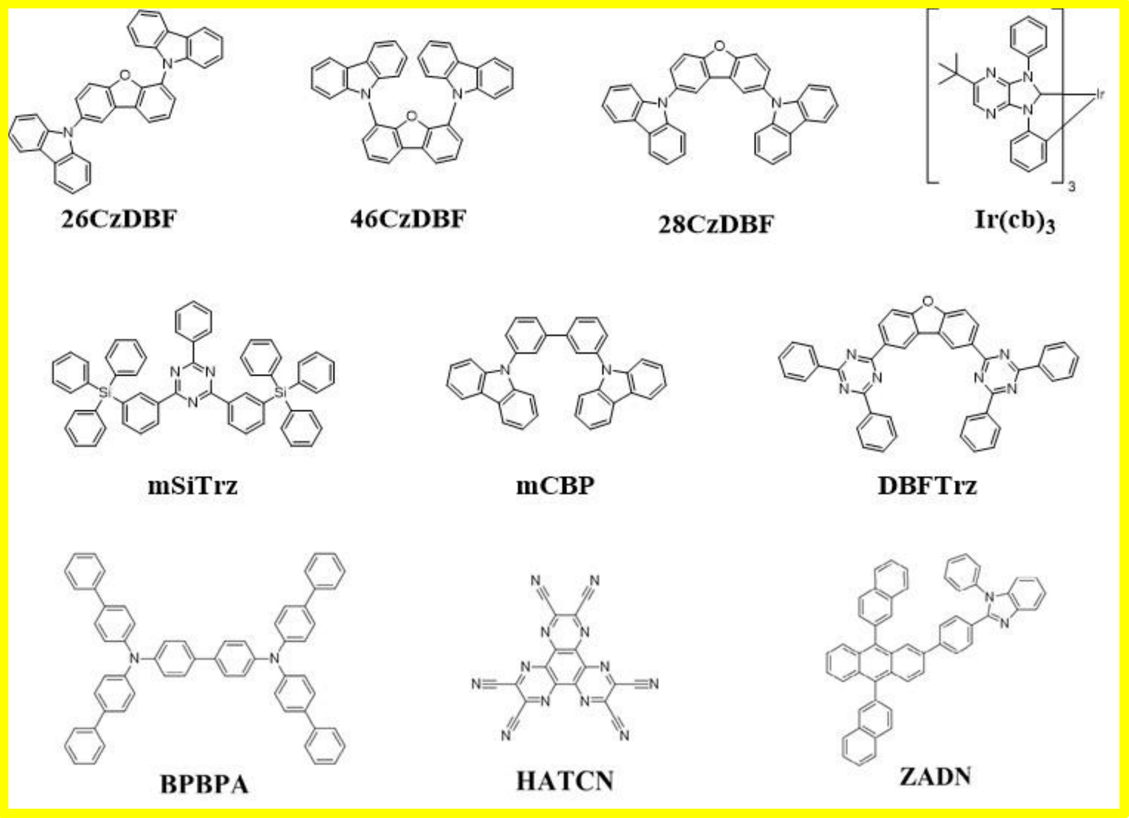

Figure S3. The chemical structures of the used materials.

References

[1] S. Jang, K. H. Lee, J. Y. Lee, Y. Lee, *J. Mater. Chem. C*, 2019, **7**, 826-834.

[2] S. H. Jeong, C. W. Seo, J. Y. Lee, N. S. Cho, J. K. Kim, J. H. Yang, *Chem. Asian J.*, 2011, **6**, 2895-2898.
